# Comprehensive analysis and reinterpretation of Cenozoic mesofossils reveals ancient origin of the snapping claw of alpheid shrimps

**DOI:** 10.1038/s41598-017-02603-5

**Published:** 2017-06-22

**Authors:** Matúš Hyžný, Andreas Kroh, Alexander Ziegler, Arthur Anker, Martin Košťák, Ján Schlögl, Adam Culka, John W. M. Jagt, René H. B. Fraaije, Mathias Harzhauser, Barry W. M. van Bakel, Andrej Ruman

**Affiliations:** 1 Department of Geology and Palaeontology, Faculty of Natural Sciences, Comenius University, Ilkovičova 6, Mlynská dolina, 842 15 Bratislava Slovakia; 2 Geological-Paleontological Department, Natural History Museum Vienna, Burgring 7, 1010 Vienna, Austria; 3 Institut für Evolutionsbiologie und Ökologie, Rheinische Friedrich-Wilhelms-Universität Bonn, An der Immenburg 1, 53121 Bonn, Germany; 4 Museu Paraense Emílio Goeldi, Campus de Pesquisa, Avenida Perimetral 1901, CEP 66077-830, Terra Firme, Belém, PA Brazil; 5 Universidade Federal de Goiás, Instituto de Ciências Biológicas, Campus Samambaia, Avenida Esperança s/n, CEP 74690-900 Goiânia, GO Brazil; 6 Institute of Geology and Palaeontology, Faculty of Science, Charles University in Prague, Albertov 6, Prague 2, 128 43 Czech Republic; 7 Institute of Geochemistry, Mineralogy and Mineral Resources, Faculty of Science, Charles University in Prague, Albertov 6, Prague 2, 128 43 Czech Republic; 8 Natuurhistorisch Museum Maastricht, De Bosquetplein 7, 6211 KJ Maastricht, Netherlands; 9 Oertijdmuseum De Groene Poort, Bosscheweg 80, 5283 WB Boxtel, Netherlands; 10 Naturalis Biodiversity Center, P.O. Box 9517, 2300 RA Leiden, Netherlands

## Abstract

Alpheid snapping shrimps (Decapoda: Caridea: Alpheidae) constitute one of the model groups for inferences aimed at understanding the evolution of complex structural, behavioural, and ecological traits among benthic marine invertebrates. Despite being a super-diverse taxon with a broad geographical distribution, the alpheid fossil record is still poorly known. However, data presented herein show that the strongly calcified fingertips of alpheid snapping claws are not uncommon in the fossil record and should be considered a novel type of mesofossil. The Cenozoic remains analysed here represent a compelling structural match with extant species of *Alpheus*. Based on the presence of several distinct snapping claw-fingertip morphotypes, the major radiation of *Alpheus* lineages is estimated to have occurred as early as 18 mya. In addition, the oldest fossil record of alpheids in general can now be confirmed for the Late Oligocene (27–28 mya), thus providing a novel minimum age for the entire group as well as the first reliable calibration point for deep phylogenetic inferences.

## Introduction

**Figure 1:**
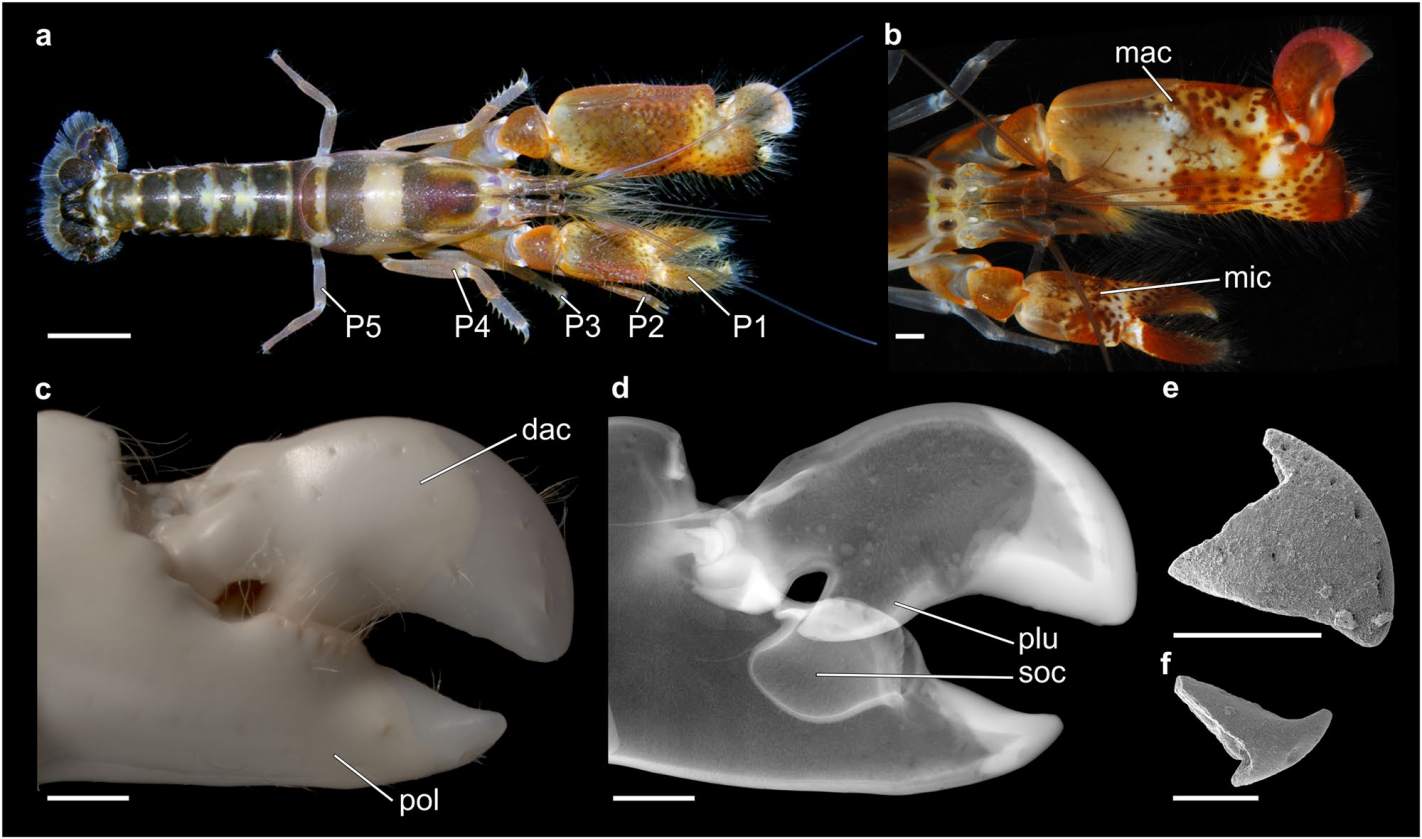
Morphology of alpheids and their snapping claw in comparison to selected fossil specimens. (**a**) Dorsal view of *Alpheus rugimanus*, in life. (**b**) Dorsal view of the assymetrical chelipeds of *Alpheus websteri*, in life. (**c**) Major cheliped of *Alpheus bisincisus*, preserved specimen (NHMW-CR-25767). (**d**) µCT-based X-ray image of the snapping claw shown in (**c**). (**e**) SEM micrograph of an allegedly cephalopod fossil specimen, the holotype of *Oligosella longi* (NCSM 10984). (**f**) SEM micrograph of another allegedly cephalopod fossil specimen, the holotype of *Oligorostra alabami* (NCSM 10980). Scale bars equal 5 mm (**a**) and 1 mm (**b**–**f**). dac = dactylus, mac = major cheliped, mic = minor cheliped, P1–P5 = pereiopods 1–5, plu = plunger, pol = pollex, soc = socket.

Alpheidae Rafinesque, 1815 (Decapoda: Caridea) is a super-diverse group of benthic marine invertebrates^1^. Species richness and ecological diversity of alpheids, popularly known as pistol or snapping shrimps, are reflected in a number of specialised behaviours. Many snapping shrimps are obligate or facultative symbionts of other marine animals, such as sponges, corals, polychaetes, bristle worms, or fishes^2–5^. In addition, some alpheids exhibit protandrous or possibly simultaneous hermaphroditism^6, 7^, while others live in groups and constitute the only known eusocial marine invertebrates^8, 9^. The most characteristic structural feature of alpheids is the snapping claw, a specialised appendage resulting from the modification of the distal-most elements of the major cheliped of a highly asymmetrical pair of first pereiopods (Fig. 1a,b). The cheliped fingers are composed of a usually dorsally located dactylus and a predominantly ventrally located pollex (Fig. 1c). During the act of snapping, the plunger (Fig. 1d), a cuticular protrusion situated on the ventral side of the dactylus, rapidly enters into a corresponding socket located on the dorsal side of the pollex (Fig. 1d). This results in the displacement of a small volume of water^10^, which is coupled with a loud cracking sound originating from the collapse of a cavitation bubble^11^. Additionally, closure of the snapping claw is accompanied by a short flash of light, a phenomenon known as shrimpoluminescence^12^. Due to their extraordinary morphological adaptations and remarkable diversity, alpheids constitute one of the model groups for studies on the evolution of complex traits among benthic marine invertebrates^3, 13, 14^.

The more recent phylogenetic inferences for Alpheidae are based on molecular analyses of selected taxa^2, 15–18^, most of them involving the two large genera *Alpheus* Fabricius, 1798 and *Synalpheus* Spence Bate, 1888, as well as on a comprehensive phylogenetic analysis of morphological characters^3^. However, reliable fossil data that would permit calibrating molecular clock estimates of alpheid evolution are still missing. This is primarily a consequence of the limited fossilization potential of the decapod cuticle^19–22^.

Despite this limitation, Kobayashi and colleagues^23^ recently suggested that small (<5 mm), isolated decapod body parts with a distinct triangular shape from the Middle Pleistocene of Japan (250–630 kya) could represent alpheid remains. More reports of similar material from coeval or younger Japanese strata followed^24–26^. In parallel, Jagt and colleagues^27, 28^ reported on similar structures from the Middle Miocene of the Netherlands (16 mya) and also interpreted these as remains of alpheid snapping shrimps. Those authors additionally mentioned occurrences from the Middle Miocene of Poland (13–15 mya) and commented upon a possible record from the Early Miocene of France (20–4 mya). These latter samples had, however, originally been interpreted as remains of cephalopods (Mollusca: Cephalopoda)^29^. The association of similarly shaped fossils with cephalopod remains was based on fragmentary material from the Late Oligocene of Alabama (27–28 mya) that was used to erect two extinct genera: *Oligosella* Ciampaglio & Weaver, 2008 (Fig. 1e) was interpreted as an embryonic stage of a representative of an unknown higher cephalopod taxon (within Coleoidea), and *Oligorostra* Ciampaglio & Weaver, 2008 (Fig. 1f) was assigned to spirulids (Cephalopoda: Spirulida)^30, 31^. However, the first interpretation of similar mesofossils was presented by Müller^32^, who associated specimens from the Early Miocene of Austria (16–17 mya) with cutting edge fragments of claws belonging to swimming crabs (Decapoda: Brachyura: Portunidae). Thus, several different taxonomic assignments of potentially highly recognizable fossil structures have been presented, but, until now, a rigorous analysis to support any of these hypotheses was lacking.

Using a set of invasive and non-invasive techniques, the present study provides the first comprehensive structural examination of these enigmatic remains. We demonstrate here that previously described as well as newly collected fossil samples not only represent remains of alpheids, but can in many cases even be identified as the fingertips of the snapping claws of early representatives of the genus *Alpheus*. These findings lead to the assignment of the oldest fossil record of alpheid shrimps to the Late Oligocene, almost 30 million years ago.

## Results

**Figure 2:**
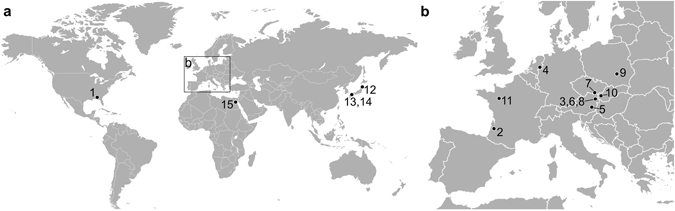
Geographical distribution of fossil specimens incorporated into this study. The numbers indicate the localities of the specimens listed in Table 1. (**a**) Localities in America, Africa, and Asia. (**b**) Localities in Europe. The maps are modified templates (http://www.freepik.com/free-vector/gray-world-map_788336.htm, https://commons.wikimedia.org/wiki/File:BlankEurope.png).

**Figure 3:**
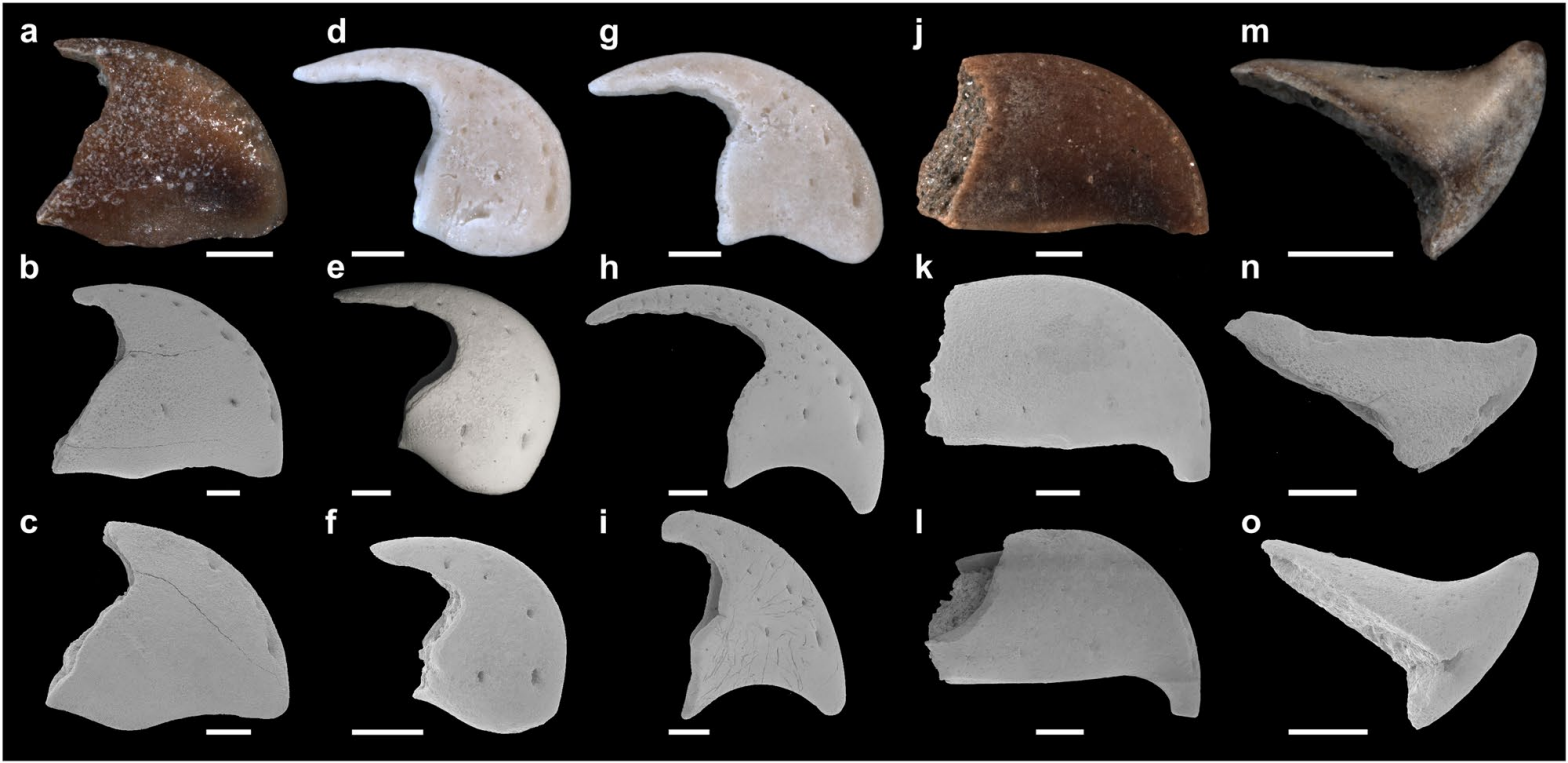
Morphology of selected fossil samples incorporated into this study. (**a**–**c**) Triangular morphotype with a short hook. (**d**–**f**) Triangular morphotype with a long hook and a convex margin. (**g**–**i**) Triangular morphotype with a long hook and a concave margin. (**j**–**l**) Almost rectangular morphotype with a blunt tip. (**m**–**o**) Morphotype with a strong blunt end. Specimens: (**a**) UMJGP 211462. (**b**) UMJGP 211458. (**c**) NHMW 2006z0343/0026. (**d**) NHMW 2016/0152/0010. (**e**) KGP-MH DU-001. (**f**) UMJGP 211446. (**g**) NHMW 2016/0152/0011. (**h**) NHMW 2016/0152/0004. (**i**) NHMW 2016/0152/0005. (**j**) UMJGP 211463. (**k**) UMJGP 211447. (**l**) UMJGP 211448. (**m**) UMJGP 211464. (**n**) UMJGP 211452. (**o**) UMJGP 211450. Upper line shows photographs, while middle and lower lines show SEM micrographs. Scale bars equal 500 µm.

Isolated, small (<5 mm) fossils from a broad geographical range, including North America, Europe, Africa, and Asia (Fig. 2) and from various stratigraphic settings (Table 1) were found to possess distinct shapes (Figs 1e,f and 3). Five morphotypes were recognised among the studied samples, with four of them relatively similar in shape: I) triangular with a short hook (Figs 1e and 3a–c); II) triangular with a long hook and a convex margin (Fig. 3d–f); III) triangular with a long hook and a concave margin (Fig. 3g–i); and IV) almost rectangular with a blunt tip (Fig. 3j–l). The fifth morphotype, with a strong blunt end, differed more substantially from the other four morphotypes (Figs 1f and 3m–o). The following aspects strongly argue for an interpretation of these fossils as remains of alpheid shrimps.

**Table 1:** Fossil specimens used in the present study.

Number	Stratigraphy	Age	Locality	Lithostratigraphy	Lithology	Specimens	Reference
1	Late Oligocene (Chattian)	27–28 mya	St. Stephens Quarry, Alabama, USA	Chickasawhay Limestone	Limestone	NCSM 10978 to NCSM 10995	30, 31
2	Early Miocene (Aquitanian)	20.4–23 mya	Saint-Morillon, Gironde, France	Plantat Fm.	Sandy clays	Private collection	27, 29
3	Early Miocene (‘Karpatian’ = late Burdigalian)	16–17 mya	Teiritzberg, Lower Austria, Austria	Korneuburg Fm.	Clays	NHMW 1997z0171/0001 to NHMW 1997z0171/0003, NHMW 2016/0155/0001	32, this study
4	Middle Miocene (early Langhian)	16 mya	Miste, Gelderland, Netherlands	Breda Fm.	Sands	MAB k.3570	27, 28
5	Middle Miocene (early ‘Badenian’ = Langhian)	15 mya	Wetzelsdorf, Styria, Austria	Florian Beds	Clays	UMJGP 75579, UMJGP 211443 to UMJGP 211468	This study
6	Middle Miocene (middle ‘Badenian’ = Langhian)	14–15 mya	Bernhardsthall, Lower Austria, Austria	Jakubov Fm.	Clays	NHMW 2016/0151/0001	This study
7	Middle Miocene (middle ‘Badenian’ = Langhian)	14–15 mya	Kienberg, Mikulov, Czech Republic	Hrušky Fm.	Sands	NHMW 2006z0343/0022 to NHMW 2006z0343/0031	This study
8	Middle Miocene (‘Badenian’ = Langhian-early Serravallian)	13.3–16 mya	Bad Vöslau, Lower Austria, Austria	Baden Fm.	Clays	NHMW 2016/0154/0001 to NHMW 2016/0154/0011	This study
9	Middle Miocene (early ‘Badenian’ = Langhian)	13–15 mya	Korytnica, Mazovia, Poland	Korytnica Clays	Clays	Radwański collection, University of Warsaw	27
10	Middle Miocene (late ‘Badenian’ = early Serravallian)	13–13.5 mya	Dubová, Pezinok, Slovakia	Studienka Fm.	Clays	KGP-MH DU-001, KGP-MH DU-002	This study
11	Late Miocene (Tortonian)	7.2–11.6 mya	Saint-Clément-de-la-Place, Maine-et-Loire, France	Redonien Chaud	Sands	NHMW 2016/0190/0001 to NHMW 2016/0190/0004	47
12	Middle Pleistocene (Ionian)	250–630 kya	Atsumi, Aichi Prefecture, Japan	Atsumi Group	Silty sands	MFM142476, MFM1424520	23, 24
13	Middle Pleistocene (Ionian)	230–250 kya	Amakusa, Kumamoto Prefecture, Japan	Ogushi Fm.	Clays	GCM-IVP3164 to GCM-IVP3167	25
14	Late Pleistocene (Tarantian)	125 kya	Minamishimabara, Nagasaki Prefecture, Japan	Oe Fm.	Sands	MFM145530, MFM145531	26
15	Late Pleistocene (Tarantian)	117–126 kya	Hurghada, al-Bahr al-Aahmar, Egypt	—	Inter-reefal sands	NHMW 2016/0152/0001 to NHMW 2016/0152/0011, NHMW 2016/0153/0001 to NHMW 2016/0153/0005	This study

Material listed in geochronological order. The consecutive numbers correspond to localities depicted in Fig. 2. GCM = Goshoura Cretaceous Museum, Amakusa, Japan; KGP = Department of Geology and Palaeontology, Comenius University, Bratislava, Slovakia; MAB = Oertijdmuseum De Groene Poort, Boxtel, Netherlands; MFM = Mizunami Fossil Museum, Mizunami, Japan; NCSM = North Carolina Museum of Natural Sciences, Raleigh, NC, USA; NHMW = Naturhistorisches Museum, Vienna, Austria; UMJGP = Department for Geology and Palaeontology, Universalmuseum Joanneum, Graz, Austria; Fm. = formation kya = thousands years ago; mya = million years ago.

**Figure 4:**
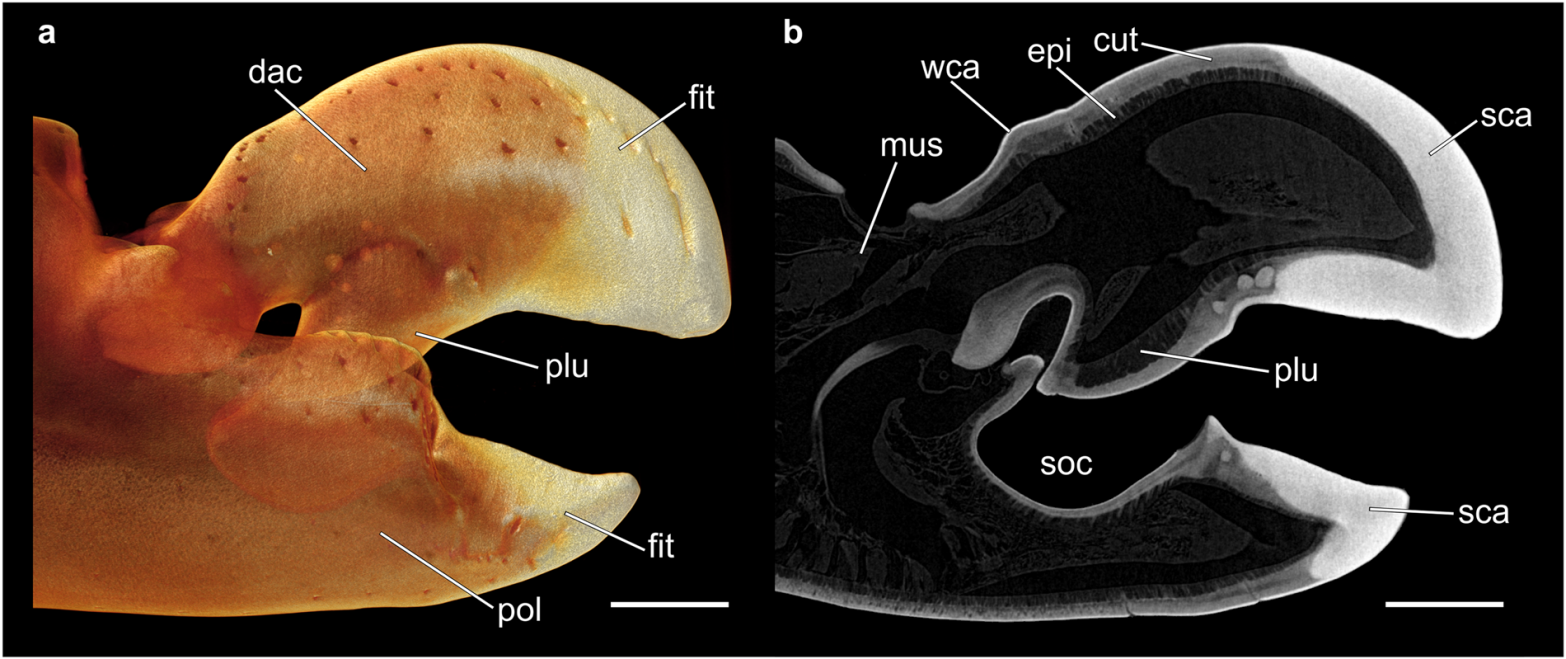
Calcification pattern of the extant alpheid snapping claw. (**a**) µCT-based volume rendering of the snapping claw of *Alpheus bisincisus*, preserved specimen (NHMW-CR-25767). (**b**) Virtual sagittal section through the same µCT dataset illustrating differences in X-ray absorption caused by different degrees of cuticular calcification. Scale bars equal 1 mm. cut = cuticle, dac = dactylus, epi = epidermis, fit = fingertip, mus = muscle, plu = plunger, pol = pollex, sca = strong calcification, soc = socket, wca = weak calcification.

**Figure 5:**
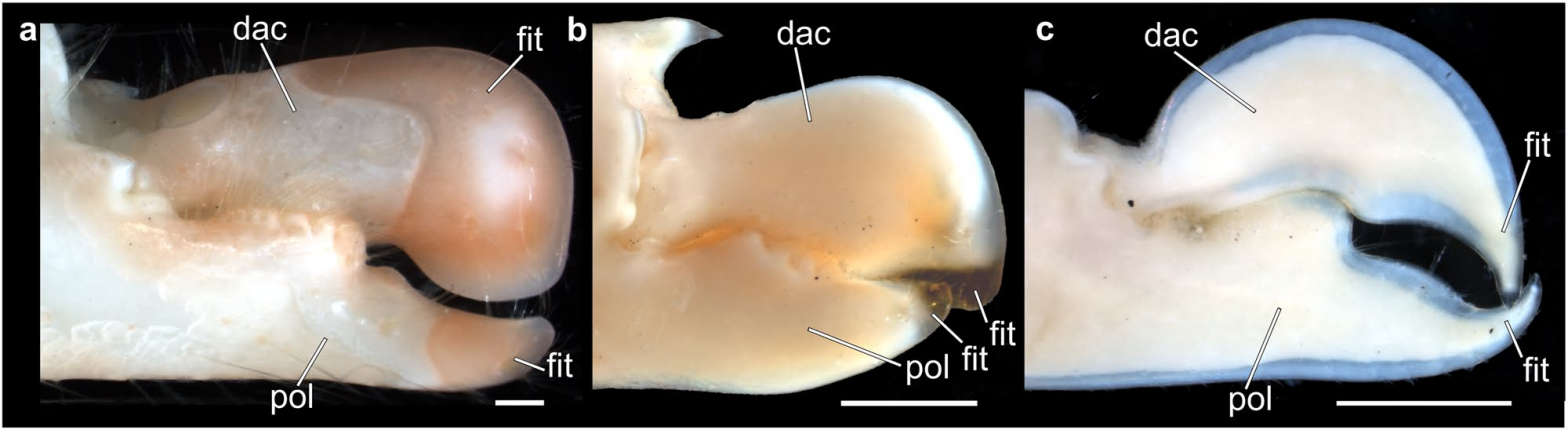
Comparative morphology of snapping claws among selected decapod taxa. (**a**) Alpheidae: *Alpheus armatus*, preserved specimen (NHMW-CR-19580). (**b**) Alpheidae: *Synalpheus gambarelloides*, preserved specimen (NHMW-CR-962). (**c**) Palaemonidae: *Coralliocaris graminea*, preserved specimen (NHMW-CR-7760). Scale bars equal 500 µm. dac = dactylus, fit = fingertip, pol = pollex.

All fossil specimens exhibited a consistent preservation pattern: they appeared to be broken off along a similar line of structural weakness. In decapods, such a line is known to mark differences in the calcification pattern between the distal tip and the remainder of the original structure^21, 22^. The particular preservation pattern observed is highly reminiscent of the claw fingertips of alpheids. For example, in all species of *Alpheus*, the fingertip of the snapping claw is always more strongly calcified than the remainder of the claw (Fig. 4), resulting in an externally identifiable boundary between these two areas (Fig. 5a). This boundary is particularly conspicuous in living individuals, in which the fingertips of the snapping claw are pale pinkish, reddish, or purplish, thereby markedly contrasting with the different colour of the rest of the claw (Fig. 1a). In contrast to *Alpheus*, all species of *Synalpheus* as well as some species of other alpheid genera (e.g., *Alpheopsis* Coutière, 1897 or *Nennalpheus* Banner & Banner, 1981) possess claw fingertips that are not calcified, but instead are corneous (i.e. proteinaceous), semi-transparent, and amber yellow in colour (Fig. 5b). The claw fingertips of the remaining alpheid taxa are similar to the rest of the claw: here, sometimes only the distal-most portion of the fingertips may be slightly more calcified or corneous than the rest of the finger. Further decapod taxa possessing a functional snapping claw, such as some representatives of the palaemonid shrimps (Decapoda: Caridea: Palaemonidae), have uncalcified fingertips that do not display a clear boundary between the fingertip and the remainder of the claw (Fig. 5c).

**Figure 6:**
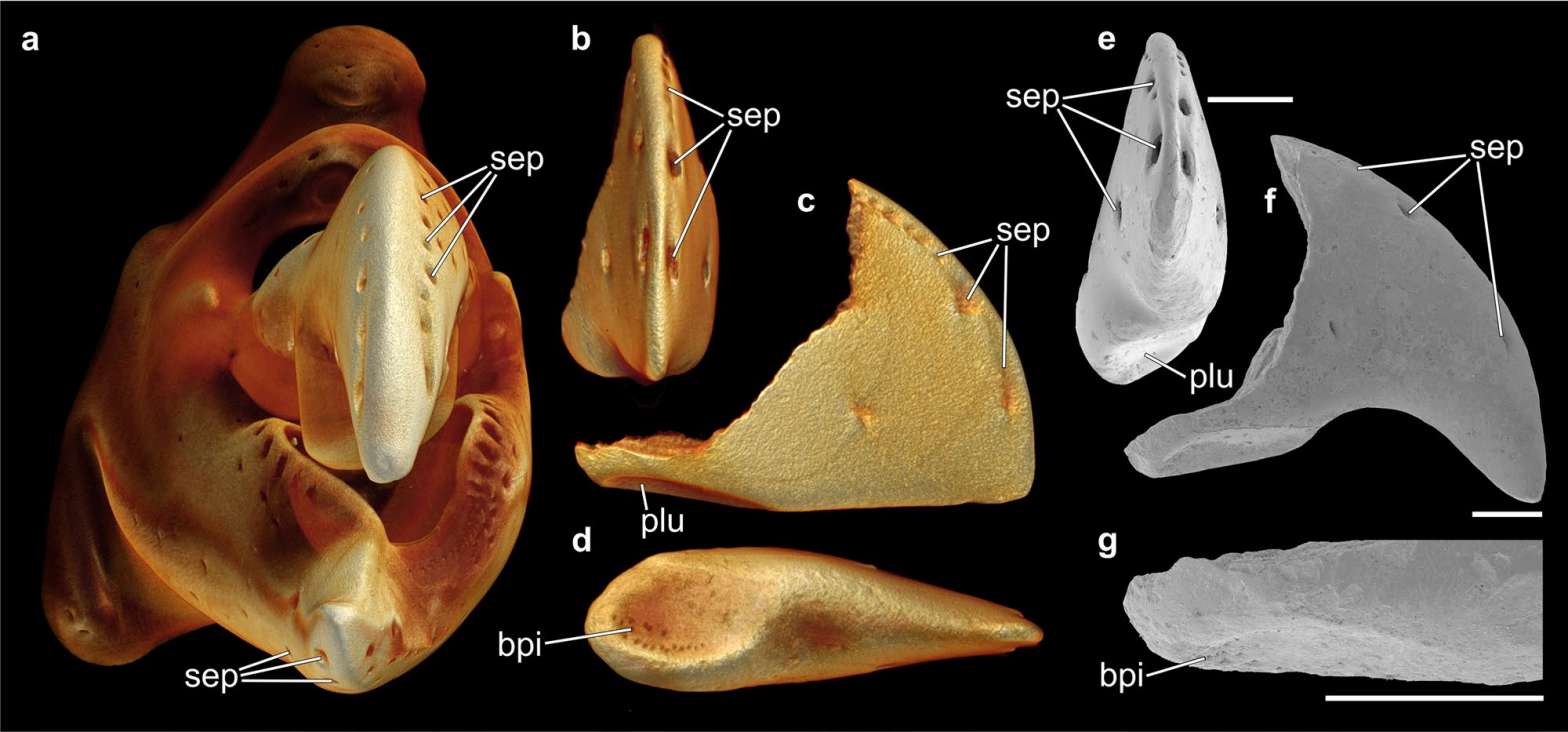
Pore arrangement in extant alpheid snapping claws and corresponding fossil samples. (**a**) Anterior view of a µCT-based volume rendering of the snapping claw of *Alpheus bisincisus*, preserved specimen (NHMW-CR-25767). (**b**–**d**) µCT-based volume renderings of a fossil dactylus fingertip (NHMW 2016/0154/0010). Anterior (**b**), inner (**c**), and occlusal (**d**) views. (**e**,**f**) SEM micrographs of a fossil dactylus fingertip (NHMW 2016/0154/0006). Anterior (**e**) and inner (**f**) views. (**g**) Close-up view of the basal pits located on the occlusal side of a fossil dactylus fingertip (NHMW 2016/0154/0004). bpi = basal pit, plu = plunger, sep = setal pore.

All fossil specimens exhibited rows of pores along their crests (Fig. 6). The arrangement of these pores was symmetrical on both sides of the fossil structures (Fig. 6b,c,e,f). Such an arrangement of pores can also be found in claw fingertips of extant representatives of *Alpheus* (Fig. 6a). Based on their size, location, and pattern of distribution, these structures were identified as setal pores. Another similarity between most of the fossil material and extant specimens of *Alpheus* was the presence of basal pits on the flattened side of the fossil structure (Fig. 6d,g). In *Alpheus*, these pits bear stamen-shaped sensillae that serve as sensory structures^33^.

**Figure 7:**
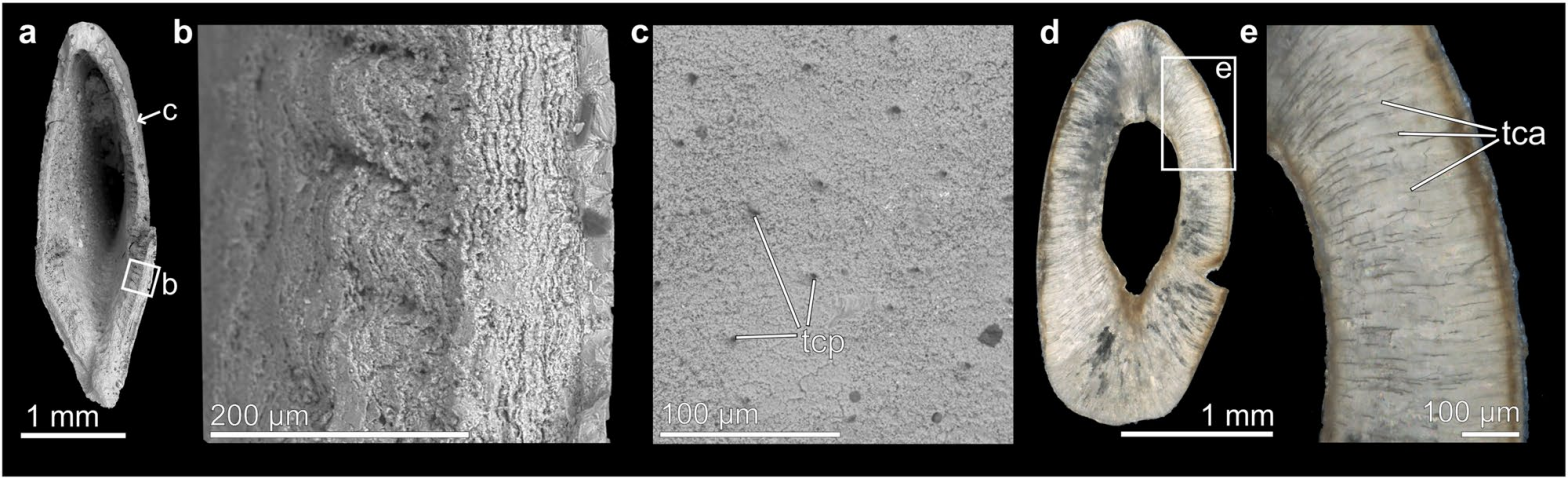
Microstructure of the fossil samples. (**a**) Posterior view of a fossil dactylus fingertip (KGP-MH DU-002) analysed using SEM. (**b**) Detail of the same specimen, showing three-fold cuticular layering. (**c**) Detail of the surface of the same specimen, showing the external pores of tegumental canals. (**d**) Thin section of a fossil dactylus fingertip (NHMW 2016/0154/0008) as seen under polarised light. (**e**) Detail of the same specimen, showing tegumental canals located inside the cuticle. tca = tegumental canal, tcp = tegumental canal pore.

Analyses of the internal structure of the fossil samples showed a distinct layering reminiscent of the cuticle found in decapods (Fig. 7a,b)^21^. SEM micrographs of the external layer revealed the presence of numerous pores (Fig. 7c), whilst thin sections showed elongated structures penetrating the different layers (Fig. 7d–e). In decapods, these so-called tegumental canals are associated with the transportation and deposition of cuticular material^21, 34^.

**Figure 8:**
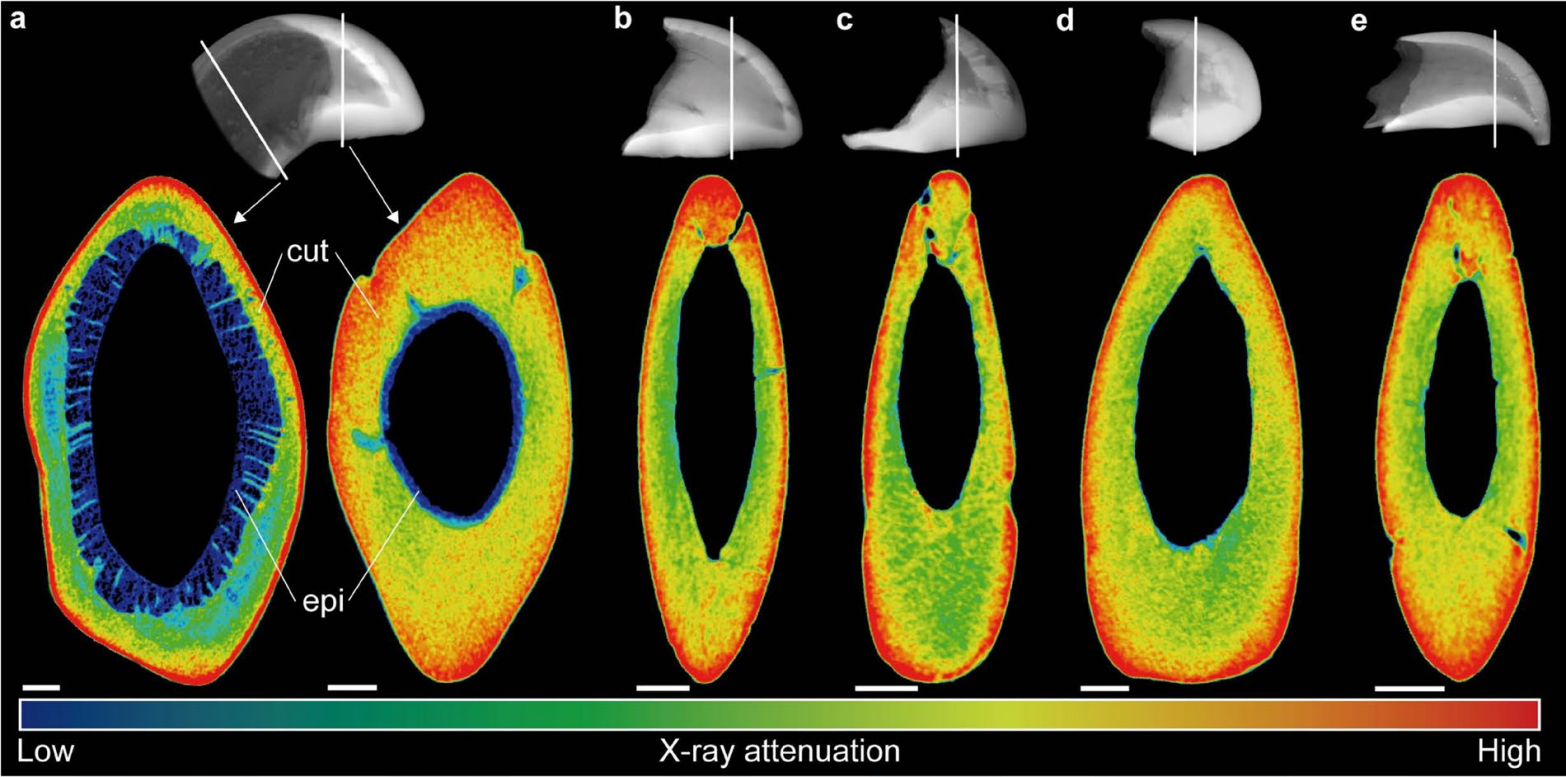
X-ray attenuation in an extant alpheid snapping claw and corresponding fossil samples. The upper line shows µCT-based X-ray images that indicate the position of the false colour-coded virtual sections depicted below. (**a**) Extant specimen: *Alpheus bisincisus* (NHMW-CR-25767). (**b**–**e**) Fossil specimens: (**b**) UMJGP 211460, (**c**) NHMW 2016/0154/0010, (**d**) NHMW 2016/0154/0009, and (**e**) UMJGP 211461. Note that in (**a**) the musculature inside the claw was virtually removed to allow for better comparison. Scale bars equal 100 µm. epi = epidermis, cut = cuticle.

Further analyses of the internal structure of extant as well as fossil claw fingertips revealed significant differences in X-ray attenuation between the proximal and distal portions of the snapping claw dactylus (Fig. 8). In the distal part, an increase in X-ray attenuation from the inside to the outside was observed that corresponded to differences in cuticular density resulting from differing degrees of calcification (Fig. 4b). The pattern of cuticular layering seen in the extant sample (Fig. 8a) was very similar to that seen in the fossil specimens (Fig. 8b–e).

**Figure 9:**
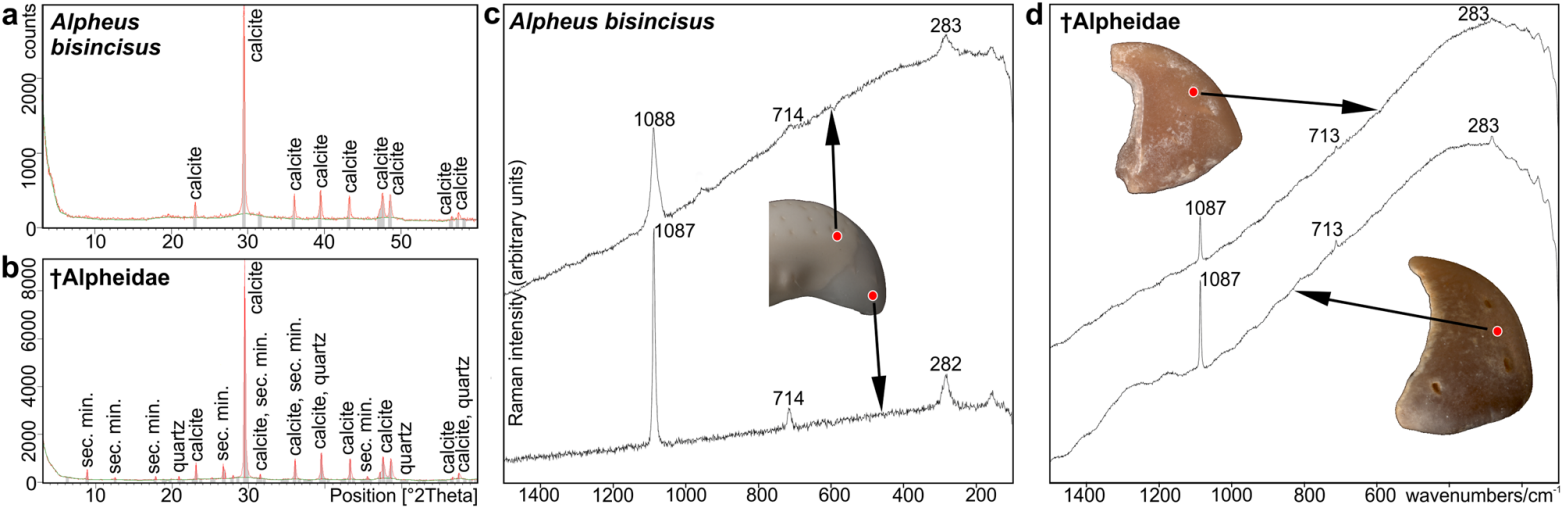
Chemical composition of extant alpheid snapping claws and corresponding fossil samples. (**a**) X-ray diffraction analysis of the claw fingertip of the extant species *Alpheus bisincisus*. (**b**) X-ray diffraction analysis of a fossil claw fingertip. (**c**) Raman spectroscopy data of the distal (upper line) and proximal parts (lower line) of the dactylus of the extant species *A*. *bisincisus*, preserved specimen (NHMW-CR-25768). (**d**) Comparative Raman spectroscopy data of two fossil dactylus fingertips (left: NHMW 2016/0154/0011, right: NHMW 2006z0343/0031).

With regard to their chemical composition, the claw fingertips of extant alpheids and those of fossil specimens were largely identical, with calcite being the principal constituent (Fig. 9a,b). The fossil samples contained a secondary substrate admixture composed of muscovite, chlorite, and quartz deriving from attached sediment particles (Fig. 9b). Results obtained using Raman spectroscopy revealed three characteristic bands of calcite at 1088/1087, 714/713, and 283/282 cm^−1^ (Fig. 9c–d). Fluorescence levels were relatively high, implying the presence of organic and inorganic impurities. Furthermore, in the proximal portion of the dactylus of the extant specimen (Fig. 9c), a major broadening of the 1088/1087 cm^−1^ calcite band was observed, which can be explained by the lower degree of calcification of the proximal portion of the alpheid snapping claw.

## Discussion

Alpheid shrimps are model organisms for studying morphological variation, one of the principal causes for adaptive radiation. In this sense, the key innovation of alpheids is the snapping claw^3^. This specialised organ is a multifunctional tool used for various inter- and intraspecific behaviours, such as aggression, warning, or defence, as well as for stunning and killing prey^9, 14^. The functional morphology of the snapping claw has been studied extensively^3, 10, 35^ and several physical phenomena associated with snapping, including water jets, light production, and cavitation bubbles have received considerable attention^11, 12, 36^. However, reliable fossil material that would permit studying the evolution of the alpheid snapping claw more comprehensively or to calibrate molecular clock estimates for alpheids in general had previously not been identified.

The poor fossil record of Alpheidae is a direct consequence of two principal factors: low fossilisation potential of small-sized decapods^19^ and difficulties in attributing fossil remains to alpheids and not to other shrimps. The latter impediment is illustrated by the interpretation of some of the fossil samples studied herein as cutting edge fragments of the claws of swimming crabs^32^ or as the rostra and beaks of cephalopods^29–31^. However, the chelipeds of derived alpheids differ morphologically from those of all other decapods and exhibit a unique combination of morphological characters not present in any other decapod taxon^3^. Therefore, based on the results presented above, the entire fossil material listed in Table 1 is here identified as the remains of the strongly calcified claw fingertips of alpheids. The material comprises several morphotypes of snapping claw fingertips, including four morphotypes attributable to the tip of the dactylus (Figs 1e and 3a–l) and one morphotype that can be assigned to the tip of the pollex (Figs 1f and 3m–o). Our data show that fingertips of alpheid snapping claws are not uncommon in the fossil record and should therefore be considered a novel type of mesofossil.

In taphonomic experiments using extant decapod material, strongly calcified claw fingertips were always retained, irrespective of the time of deployment^20, 22^. Due to their increased level of calcification, claw fingertips are therefore present in most fossil decapod assemblages^21^. Correspondingly, we observed a fundamental difference in the microstructure of alpheid claw fingertips in comparison to the remainder of the claw (Figs 7 and 8), as well as in the chemical composition of the respective parts (Fig. 9). These structural differences are conducive to the preferential preservation of the distal-most parts of the claw. However, a positive bias towards fossilisation of larger-sized alpheid species that possess distally strongly calcified snapping claws - primarily species of *Alpheus* - can be expected.

In fact, some of these fossil forms have morphological analogues among extant species of *Alpheus*. For instance, the large triangular dactylus with a long hook and a convex margin documented from the Pleistocene of Egypt (Fig. 3d) as well as the Miocene of Slovakia (Fig. 3e) and Austria (Fig. 3f) is an almost perfect match for the homologous structure in the extant species *Alpheus armatus* Rathbun, 1901 (Fig. 5a). In addition, the fossil triangular morphotype with a short hook (Fig. 3a–c) can be matched with the dactylus of extant representatives of the *Alpheus brevirostris* (Olivier, 1811) species group, such as *Alpheus bellulus* Miya & Miyake, 1969^36^. The co-occurrence of these forms at some localities suggests that the group was already diversifying in the Middle Miocene. As was previously shown, the co-occurrence of several, more or less closely related species of *Alpheus* at a single site is very common in modern shallow marine habitats, especially in the tropics^4, 37, 38^.

However, some of the morphological variation observed may also be attributable to differences in the degree of usage of the claw as well as the individuals’ age or sex. In general, sexual dimorphism in alpheids can be assessed by observing the more or less pronounced differences in claw shape and size among sexually mature adults^3, 38^. However, a critical evaluation of intraspecific variation in the morphology of snapping claws of extant taxa would have to be carried out first in order to determine whether or not the fossil alpheid material may be attributed to particular lineages within *Alpheus*. Nevertheless, it can be stated with confidence that already by 30 mya alpheids developed several of the snapping claw morphologies that can be observed today.

The tree topology resulting from a broad phylogenetic analysis of alpheid morphological characters suggested a single origin of the snapping claw^3^. However, the apparent parallel evolution of the snapping claw and orbital hoods – protrusions of the carapace which protect the shrimp’s eyes from mechanical damage resulting from snapping – a hypothesis first postulated by Coutière^39^, may have resulted in an interdependence of several, possibly homoplasious characters. In general, many conspicuous features of alpheid claws appear prone to convergent evolution^3^, and the snapping mechanism may not be an exception. Structural differences between the snapping claws of *Alpheus* and *Synalpheus* ^3^ (Fig. 5a,b) as well as preliminary molecular analyses^16^ support multiple origins of this highly specialised appendage, a situation also occurring in palaemonid shrimps^40^.

Using a molecular clock approach, the origin of Alpheidae was previously estimated to around 150 mya^41^. Earlier investigations of a selection of American species of *Synalpheus* ^2^ found evidence for a major radiation of this taxon during the Late Miocene/Early Pliocene (5–7 mya), i.e. prior to the final closure of the Isthmus of Panama^42^. In addition, Hurt and colleagues^17^ concluded that at least two transisthmian species pairs of *Alpheus* diverged well before the final closure of the Isthmus of Panama, one of them possibly as early as 13 mya. The split between the most divergent transisthmian pairs of *Alpheus* was therefore estimated to have occurred during the Early Miocene at about 18 mya^43^, which is corroborated with the present observations of several distinct claw fingertip morphotypes from Middle Miocene deposits (Table 1, Fig. 3a–f). However, as shown here, the emergence of a complex snapping claw must have taken place much earlier, at least prior to the Late Oligocene: the oldest known fossil alpheid samples originate from the Chickasawhay Limestone (Table 1; Fig. 1e,f), a unit dated at 27–28 mya^44, 45^. This date is more than ten^27, 28^ or even more than 25 million years^23–26^ older than the previous, uncertain records of alpheid fossil remains. Our data thus provide the first reliable calibration points for future phylogenetic inferences focusing on the evolution of complex behavioural and morphological traits among one of the principal model taxa of benthic marine invertebrates.

## Methods

### Specimens

Fossil specimens extracted from bulk samples that had been processed wet through a stack of sieves were manually picked from washed residues under a binocular. Detailed information on fossil specimens is provided in Table 1, while the extant specimens used in the present study were: *Alpheus armatus* Rathbun, 1901 (NHMW-CR-19580), *Alpheus bisincisus* De Haan, 1849 (NHMW-CR-25767 to NHMW-CR-25771), *Alpheus rugimanus* A. Milne-Edwards, 1878 (unvouchered), *Alpheus websteri* Kingsley, 1880 (unvouchered), *Coralliocaris graminea* (Dana, 1852) (NHMW-CR-7760), and *Synalpheus gambarelloides* (Nardo, 1847) (NHMW-CR-962).

### Micro-Photography

Fossil and extant specimens were photographed using a SteREO Discovery.V20 stereomicroscope equipped with a digital camera (Carl Zeiss Microscopy, Jena, Germany).

### Scanning electron microscopy

Imaging of fossil and extant samples was carried out under high-vacuum settings using JSM-6610LV and JSM-6380LV (JEOL, Akishima, Japan) as well as S-3700N (Hitachi, Tokyo, Japan) SEMs.

### Micro-computed tomography

The entire snapping claw of one extant specimen (*Alpheus bisincisus* NHMW-CR-25767) and the claw fingertips of four fossil specimens (NHMW 2016/0154/0009, NHMW 2016/0154/0010, UMJGP 211460, UMJGP 211461) were analysed using a SkyScan 1272 µCT scanner (Bruker microCT, Kontich, Belgium). The dry specimens were placed in conical plastic tubes and scanned in air. Scanning parameters were: 60 kV source voltage, 166 µA source current, 3 µm isotropic voxel resolution, 1,706 ms exposure, 0.5° rotational steps over 180°, 2 averages, 0.25 mm aluminium filter, and 56 min scan time.

### Light microscopy

Selected specimens were manually ground to a thin slice. After transfer to a glass slide and fine grinding to the target plane (75 µm), sections were polished and observed under a SteREO Discovery.V20 stereomicroscope using polarising filters.

### X-ray diffraction

Two fossil specimens and one sample of an extant taxon (*Alpheus bisincisus*) were homogenised and analysed using an X’PertProMPD X-ray diffractometer (PANalytical B.V., Almelo, Netherlands). The data were processed using the commercial software X’PertHighScore 1.0d.

### Raman spectroscopy

Samples of two fossil specimens (NHMW 2006/0343/0031, NHMW 2016/0154/0011) and one extant taxon (*Alpheus bisincisus* NHMW-CR-25768) were analysed using an InVia Raman spectroscope (Renishaw, Wotton-under-Edge, United Kingdom). Excitation was provided through the 785 nm line of a diode laser. Spectra were recorded at 0.5 to 5% laser power over a spectral range of 100–1500 cm^−1^. Scanning parameters were: 20 s accumulation time, laser power < 5 mW (to avoid thermal degradation and detector saturation due to fluorescence), and 10–20 scans (to improve signal-to-noise ratio).

### Data Availability

Digital raw data have been deposited in MorphoBank under project number 2524 and are available for download^46^. Please select the ‘Media’ and ‘Documents’ tabs to access photographs, SEM micrographs, X-ray imagery, µCT image stacks, and X-ray diffraction as well as Raman spectroscopy data.

## References

[1] De Grave S, Fransen CHJM (2011). Carideorum catalogus: the recent species of the dendrobranchiate, stenopodidean, procarididean and caridean shrimps (Crustacea: Decapoda). Zool. Med. Leiden.

[2] Morrison CL, Ríos R, Duffy JE (2004). Phylogenetic evidence for an ancient rapid radiation of Caribbean sponge-dwelling snapping shrimps (*Synalpheus*). Mol. Phylogenet. Evol..

[3] Anker A, Ahyong ST, Noël PY, Palmer AR (2006). Morphological phylogeny of alpheid shrimps: parallel preadaptation and the origin of a key morphological innovation, the snapping claw. Evolution.

[4] Anker A (2012). Notes on the Indo-West Pacific shrimp genus *Athanopsis* Coutière, 1897 (Crustacea, Decapoda, Alpheidae), with the description of a new species associated with echiurans (Annelida, Thalassematidae). Zootaxa.

[5] Hurt C, Silliman K, Anker A, Knowlton N (2013). Ecological speciation in anemone-associated snapping shrimps (*Alpheus armatus* species complex). Mol. Ecol..

[6] Gherardi F, Calloni C (1993). Protandrous hermaphroditism in the tropical shrimp *Athanas indicus* (Decapoda: Caridea), a symbiont of sea urchins. J. Crust. Biol..

[7] Anker A (2011). Two new species of *Salmoneus* Holthuis, 1955 with a deep dorsal depression on the carapace (Crustacea, Decapoda, Alpheidae). Zootaxa.

[8] Duffy JE, Morrison CL, Ríos R (2000). Multiple origins of eusociality among sponge-dwelling shrimps (*Synalpheus*). Evolution.

[9] Duffy JE, Morrison CL, Macdonald KS (2002). Colony defense and behavioral differentiation in the eusocial shrimp *Synalpheus regalis*. Behav. Ecol. Sociobiol..

[10] Volz P (1938). Studien über das “Knallen” der Alpheiden. Z. Morphol. Oekol. Tiere.

[11] Versluis M, Schmitz B, von der Heydt A, Lohse D (2000). How snapping shrimp snap: through cavitating bubbles. Science.

[12] Lohse D, Schmitz B, Versluis M (2001). Snapping shrimp make flashing bubbles. Nature.

[13] Nakashima Y (1987). Reproductive strategies in a partially protandrous shrimp, *Athanas kominatoensis* (Decapoda, Alpheidae): sex change as the best of a bad situation. J. Ethol..

[14] Schmitz B, Herberholz J (1998). Snapping behaviour in intraspecific agonistic encounters in the snapping shrimp (*Alpheus heterochaelis*). J. Biosci..

[15] Williams ST, Knowlton N (2001). Mitochondrial pseudogenes are pervasive and often insidious in the snapping shrimp genus. Alpheus. Mol. Biol. Evol..

[16] Bracken, H. D., De Grave, S. & Felder, D. L. Phylogeny of the infraorder Caridea based on mitochondrial and nuclear genes (Crustacea: Decapoda) In *Decapod Crustacean Phylogenetics* (eds Martin, J. W., Crandall, K. A. & Felder, D. L.) 281–305 (CRC Press, Taylor & Francis Group, 2009).

[17] Hurt C, Anker A, Knowlton N (2009). A multilocus test of simultaneous divergence across the Isthmus of Panama using snapping shrimp in the genus. Alpheus. Evolution.

[18] Hultgren KM, Hurt C, Anker A (2014). Phylogenetic relationships within the snapping shrimp genus. Synalpheus. Mol. Phylogenet. Evol..

[19] Plotnick RE (1986). Taphonomy of a modern shrimp: implications for the arthropod fossil record. Palaios.

[20] Stempien JA (2005). Brachyuran taphonomy in a modern tidal-flat environment: preservation potential and anatomical bias. Palaios.

[21] Waugh DA, Feldmann RM, Schroeder AM, Mutel MHE (2006). Differential cuticle architecture and its preservation in fossil and extant *Callinectes* and *Scylla* claws. J. Crust. Biol..

[22] Mutel MHE, Waugh DA, Feldmann RM, Parsons-Hubbard KM (2008). Experimental taphonomy of *Callinectes sapidus* and cuticular controls on preservation. Palaios.

[23] Kobayashi N, Goda T, Ohira N, Karasawa H (2008). New records of crabs and barnacles (Crustacea: Decapoda and Cirripedia) from the middle Pleistocene Atsumi Group of Aichi Prefecture, Japan. Bul. Mizunami Fossil Mus..

[24] Karasawa H, Kobayashi N, Goda T, Ohira N, Ando Y (2014). A diversity for crabs (Decapoda) from the middle Pleistocene Atsumi Group, Japan. Bul. Mizunami Fossil Mus..

[25] Ando Y, Kawano S, Ugai H (2015). Fossil stomatopods and decapods from the upper Pleistocene Ogushi Formation, Kyushu, Japan. N. Jahrb. Geol. Paläontol. Abh..

[26] Ando Y, Kawano S, Komatsu T, Niitani M (2016). Decapod crustaceans from the Pleistocene Oe Formation in Minamishimabara City, Nagasaki Prefecture, Japan. J. Fossil Res..

[27] Jagt JWM, Verschueren S, Fraaije RHB, van Bakel BWM (2015). Miocene pistoolgarnalen (Alpheidae) uit Winterswijk-Miste: wie heeft er toevallig nog liggen?. Afzettingen WTKG.

[28] Jagt JWM, Fraaije RHB, van Bakel BWM (2016). Kreeftachtigen (Ostracoda, Thoracica, Caridea, Axiidea, Anomura en Brachyura) van Winterswijk-Miste. Afzettingen WTKG.

[29] Cluzaud, A., Lesport, F., Cahuzac, B. & Janssen, A. Mollusques In *Stratotype Aquitanien* (ed. Londeix, L.) 223–232 (Publications Scientifiques du Muséum, Biotope Éditions, 2014).

[30] Ciampaglio CN, Weaver PG (2008). Two new genera of Coleoidea from the Chickasawhay Limestone (Oligocene) of Alabama. N. Jb. Geol. Paläontol. Abh..

[31] Weaver PG, Ciampaglio CN, Chandler RE (2010). An overview of coleoid cephalopods from Paleogene and Neogene aged rocks of Southern North America. Ferrantia.

[32] Müller P (1998). Decapode Crustacea aus dem Karpat des Korneuburger Beckens (Unter-Miozän, Niederösterreich). Beitr. Paläontol..

[33] Sullivan, J. & Schmitz, B. The mechanosensory system of snapper and pincer claw in snapping shrimp (*Alpheus heterochaelis*) In *Proceedings of the 25th Göttingen Neurobiology Conference*, *1997*. Volume 2 (eds Elsner, N. & Wässle, H.) 250 (Thieme, 1997).

[34] Compère, P., Jeuniaux, C. & Goffinet, G. The integument: morphology and biochemistry In *Treatise on Zoology – Anatomy*, *Taxonomy*, *Biology: The Crustacea*. Volume 1 (eds Forest, J, von Vaupel Klein, J. C. & Schram, F. R.) 59–144 (Brill, 2004).

[35] Ritzmann RE (1973). Snapping behavior of the shrimp *Alpheus californiensis*. Science.

[36] Hess D, Brücker C, Hegner F, Balmert A, Bleckmann H (2013). Vortex formation with a snapping shrimp claw. PLoS ONE.

[37] Banner AH, Banner DM (1981). Decapod Crustacea: Alpheidae. Mémoires ORSTOM.

[38] Banner DM, Banner AH (1982). The alpheid shrimp of Australia Part III: the remaining alpheids, principally the genus *Alpheus*, and the family Ogyrididae. Rec. Aust. Mus..

[39] Coutière H (1899). Les “Alpheidae”, morphologie externe et interne, formes larvaires, bionomie. Ann. Sci. Nat. Zool..

[40] Bruce AJ (1994). A Synopsis of the Indo-West Pacific genera of the Pontoniinae (Crustacea: Decapoda: Palaeomonidae). Theses Zool..

[41] Bracken HD, De Grave S, Toon A, Felder DL, Crandall KA (2010). Phylogenetic position, systematic status, and divergence time of the Procarididea (Crustacea: Decapoda). Zool. Scripta.

[42] O’Dea A (2016). Formation of the Isthmus of Panama. Sci. Adv..

[43] Knowlton N, Weigt LA (1998). New dates and new rates for divergence across the Isthmus of Panama. Proc. R. Soc. Lond. B.

[44] Siesser WG (1983). Paleogene calcareous nannoplankton biostratigraphy: Mississippi, Alabama, and Tennessee. Bull. Miss. Dept. Nat. Resources.

[45] Gradstein, F. M., Ogg, J. G., Schmitz, M. D. & Ogg, G. M. *The Geologic Time Scale* 2012. Volume 2 (Elsevier, 2012).

[46] Hyžný, M. *et al*. Comprehensive analysis and reinterpretation of Cenozoic mesofossils reveals ancient origin of the snapping claw of alpheid shrimps. Morphobank Project 2524 http://morphobank.org/permalink/?P2524 (2017). 10.1038/s41598-017-02603-5 PMC5481430 28642499

[47] Van Dingenen F, Ceulemans L, Landau BM, da Silva CM (2015). The family Nassariidae (Gastropoda: Buccinoidea) from the late Neogene of northwestern France. Cainozoic Res..

